# Universal CAR-T Cell Therapy for Cancer Treatment: Advances and Challenges

**DOI:** 10.32604/or.2025.067445

**Published:** 2025-10-22

**Authors:** Jianan Lei, Zhuona Ni, Ruidi Zhang

**Affiliations:** School of Basic Medical Sciences, Zhejiang Chinese Medical University, Hangzhou, 310053, China

**Keywords:** Chimeric antigen receptor, cancer, universal cell therapy, hematological malignancies, solid tumor

## Abstract

This review aims to explore the development, challenges, and future directions of UCAR cell therapy as a scalable alternative to autologous CAR-T for cancer treatment. Consequently, limitations of autologous CAR-T, including long production, variable quality, and cost, drive off-the-shelf UCAR development to standardize manufacturing and improve access. Current UCAR-T cell strategies focus on mitigating the risks of graft-vs.-host disease and host-vs.-graft rejection through advanced gene editing technologies, including clustered regularly interspaced short palindromic repeat-associated system Cas9-mediated knockout of the T cell receptor, human leukocyte antigen, and cluster of differentiation 52 (CD52*)*. Beyond conventional T cells, cell types such as double-negative T cells, γδT cells, and virus-specific T cells are being engineered with CARs to improve tumor targeting and minimize off-tumor toxicity. UCAR-T therapy is frequently used for hematologic malignancies, including acute lymphoblastic leukemia, non-Hodgkin lymphoma, and multiple myeloma, with efficacy and safety supported by numerous clinical studies. Although trials for solid tumors (e.g., CYAD-101, CTX130) show modest responses, challenges such as tumor heterogeneity and T cell exhaustion remain. Future research should focus on optimizing gene editing precision, integrating combination therapies, and advancing scalable manufacturing platforms. With expanded targets and cell types, UCAR therapies show promise for both hematologic and solid tumors, reshaping cancer treatment and patient outcomes.

## 1 Introduction

Chimeric antigen receptor (CAR)-T cell therapy was first proposed in 1989. This chimeric receptor provides T cells with antibody-like specificity and can effectively transmit signals that activate T cells to perform their effector functions, representing a revolutionary advance in the field of cancer immunotherapy [1,2]. This innovative approach involves the genetic engineering of a patient’s T cells to express CARs on their surface. CARs are designed to recognize and bind to specific antigens present on the surface of cancer cells, thereby enabling T cells to identify and eliminate them with high precision [3]. CAR-T cell therapy typically begins with the collection of a patient’s T cells, usually from peripheral blood [4]. These cells are then genetically modified in the laboratory to express CARs. CARs are constructed by combining a portion of the T cell receptor (TCR) that recognizes the tumor antigen with an immunoglobulin (Ig) domain, allowing T cells to bind to the antigen [5]. T cell redirection involves genetic modification with the CAR or the use of recombinant proteins designated as bispecific T cell engagers (BiTEs) [5]. The modified T cells are then expanded in culture to increase their number before being infused back into the patient [6]. CAR-T cells are designed to activate and proliferate upon encountering target antigens, leading to the destruction of cancer cells. This process is highly specific because CARs are tailored to recognize particular antigens expressed by tumor cells. This specificity minimizes the risk of off-target effects, which can cause damage to healthy cells [7,8]. The development of CAR-T cell therapy has been particularly successful in treating hematological malignancies, such as acute lymphoblastic leukemia (ALL), non-Hodgkin lymphoma (NHL), and multiple myeloma, with overall response rates (ORRs) of 44%–91% and complete response (CR) rates of 28%–68% [9]. However, the application of CAR-T cell therapy to solid tumors remains a significant challenge because these tumors often have complex antigen expression patterns and an immunosuppressive tumor microenvironment, which can hinder the efficacy of CAR-T cells [10,11]. Despite these challenges, ongoing research and development efforts have focused on improving the efficacy, safety, and accessibility of CAR-T cell therapy. These include the development of novel CAR designs, optimization of manufacturing processes, and exploration of combination therapies with other immunotherapies and targeted therapies. The goal is to make CAR-T cell therapy a viable treatment option for a wider range of patients with cancer, including those with solid tumors [12]. Mesothelin (MSLN) has become a new immune target, employing regional routes of delivery, introducing novel modifications leading to enhanced tumor infiltration and persistence, and improved safety profiles and combining anti-MSLN CAR-T cells with standard therapies, which could render them more efficacious in the treatment of solid malignancies [13,14].

Traditional autologous CAR-T cell therapy has complex production and cost limitations that are not conducive to its widespread popularization. In this context, the systematic advantage of replacing autologous CAR-T cells with universal chimeric antigen receptor-T (UCAR-T) cells is obvious [15]. UCAR-T cell therapy can be produced in batches at lower costs and without the need to wait for individual patients. This review explores its concept, development, and application in cancer treatment, with particular attention to its underlying mechanisms, therapeutic advantages, and existing challenges. We compare UCAR-T with conventional CAR-T therapies in terms of biological mechanisms, manufacturing processes, and clinical applications. Additionally, we examine its current research progress and clinical status across multiple cancer types, analyze key technical, safety, and ethical concerns, and discuss potential strategies to overcome these limitations. Ultimately, this study aims to support the advancement of UCAR-T cell therapy, stimulate further research, and improve clinical outcomes for cancer patients.

## 2 Comparison of CAR-T and UCAR-T Cell Therapies

### 2.1 Mechanism of CAR-T Cell Therapy

#### 2.1.1 CAR Structure and Function

The CAR cell surface receptor is a synthetic protein that interacts with traditional T cell activation. CAR activation of T cells usually does not require major histocompatibility complex (MHC)-peptide binding to T cell surface antigens via antigen-presenting cells, but activates T cells directly. The structure of CARs plays a crucial role in the efficacy and specificity of CAR-T cell therapy [16]. This therapy involves genetic modification of T cells to express tumor-specific receptors, enabling T cells to produce a tumor-specific immune response when returned to the patient [17]. CAR usually consists of four domains: the extracellular antigen-binding domain, spacer or hinge region, transmembrane domain, and intracellular signaling domain [18].

The extracellular domain is responsible for recognizing and binding tumor-associated antigens (TAAs) expressed on the surface of cancer cells [19]. This domain is typically derived from an antibody that provides high affinity and specificity for a target antigen. The intracellular signaling domain transmits signals from the antigen-binding domain to T cells, leading to T cell activation and subsequent tumor cell death [11]. This domain is often derived from TCR or cluster of differentiation 28 (CD28)/4-1BB co-stimulatory molecules, which activate T cells and enhance their effector functions. The antigen-binding domain of CARs can be engineered to target a wide range of TAAs, allowing for the development of CAR-T cell therapies for various types of cancer. For example, CARs targeting cluster of differentiation 19 (CD19), a protein expressed on the surface of B-cell malignancies, have been successfully used to treat patients with relapsed or refractory B-cell acute lymphoblastic leukemia (B-ALL) and NHL [20]. Additionally, CARs targeting B cell maturation antigen (BCMA), a protein expressed on multiple myeloma cells, have demonstrated encouraging outcomes in clinical studies for the treatment of this hematologic malignancy. The intracellular signaling domain of CARs is also a critical factor in determining the antitumor activity of CAR-T cell therapy [21]. Early generations of CARs contained only the TCR signaling domain, which is associated with limited efficacy and increased toxicity. To address this issue, subsequent generations of CARs have incorporated costimulatory domains, such as CD28 or 4-1BB, to enhance T cell activation and improve the therapeutic response [22]. This has resulted in improved efficacy and reduced toxicity in clinical trials. The hinge region of the CARs serves as a flexible linker between the extracellular and intracellular domains. This region allows for conformational changes in the CAR, which are important for antigen binding and T cell activation [23]. The hinge region can also be engineered to improve CAR stability and reduce the risk of CAR internalization and shedding, leading to antigen loss and reduced efficacy [24]. Ongoing research is focused on optimizing the design of CARs to enhance their specificity, affinity, and stability as well as to reduce toxicity and improve the therapeutic response in patients with various types of cancer [25].

#### 2.1.2 Food and Drug Administration (FDA) Approval and Commercialization of Traditional CAR-T

The FDA approval of CAR-T cell therapies has marked significant milestones in the development and commercialization of this innovative cancer treatment. Since the first approval of Kymriah (Tisagenlecleucel) for the treatment of B-ALL in 2017 [26], the FDA has approved several CAR-T cell therapies targeting various hematologic malignancies. These approvals include Yescarta (Axicabtagene Ciloleucel), which is used for the treatment of relapsed/refractory large B cell lymphoma (r/r LBCL) and relapsed/refractory follicular lymphoma [27]; Tecartus (Brexucabtagene Autoleucel), which is used to treat relapsed/refractory mantle cell lymphoma and relapsed/refractory precursor B cell acute lymphocyte leukemia [28]; Breyanzi (Lisocabtagene Maraleucel), which is used to treat r/r LBCL [29]; ABECMA (Idecabtagene vicleucel) for r/r multiple myeloma who have had at least four prior therapies [30]; Carvykti (Ciltacabtagene autoleucel) for r/r multiple myeloma who have had at least four prior therapies [31]; and Aucatzyl (Obecabtagene Autoleucel), which is used to treat adult patients with relapsed/refractory precursor B cell acute lymphocyte leukemia [32]. These approvals have not only validated the efficacy of CAR-T cell therapy but have also paved the way for its commercialization. The commercialization of CAR-T cell therapy is a complex process that involves various challenges [33]. One primary challenge is the high cost of manufacturing CAR-T cells [34]. The process of isolating, engineering, and expanding T cells *ex vivo* is time-consuming and requires specialized facilities and skilled personnel. This has led to the use of high-priced tags for CAR-T cell therapies, making them accessible to only a limited number of patients. However, efforts are being made to optimize manufacturing processes and reduce costs, thereby making CAR-T cell therapies more affordable [35]. Another challenge in the commercialization of CAR-T cell therapies is the complexity of regulatory approval [36]. The FDA approval process for CAR-T cell therapies is rigorous and requires extensive preclinical and clinical data to demonstrate safety and efficacy [37]. This process can be time-consuming and expensive, further delaying the availability of these therapies to patients. Despite these challenges, the commercialization of CAR-T cell therapies has led to significant advancements in the treatment of hematologic malignancies [38]. The success of CAR-T cell therapies has sparked considerable interest in the development of UCAR-T cell therapies, which aim to overcome the challenges associated with current CAR-T cell therapies, such as finite production capacity and the need for personalized treatment. The development of UCAR-T cell therapies could potentially make this innovative cancer treatment more accessible and affordable for a broader patient population.

### 2.2 Why Develop UCAR-T Cell Therapy?

#### 2.2.1 Mechanism of UCAR-T Cell Therapy

**Figure 1:**
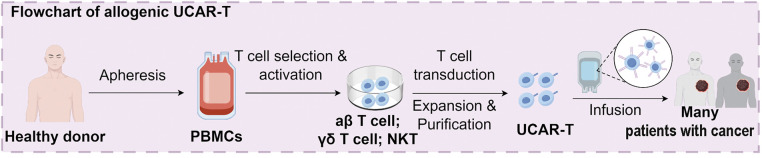

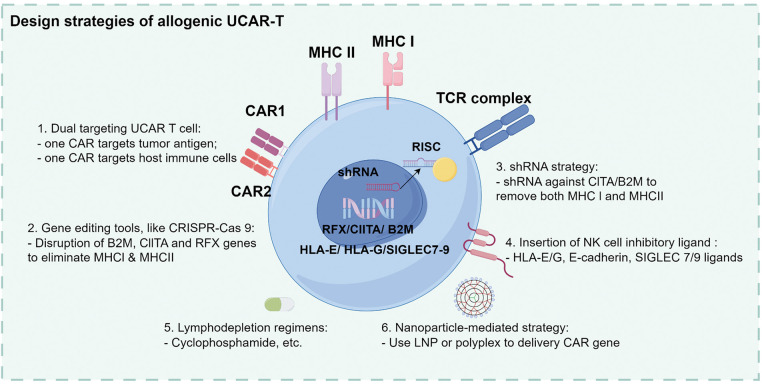
Overview of the manufacturing and design strategies for allogeneic UCAR-T cells. UCAR-T cells are derived from healthy donor peripheral blood mononuclear cells (PBMCs) through T cell selection, activation, CAR transduction, and *in vitro* expansion. Design strategies include dual-targeting CARs (tumor and host immune cells), gene editing using CRISPR-Cas9 (targeting B2M, CIITA, and RFX), shRNA-mediated silencing, insertion of NK cell inhibitory ligands (e.g., HLA-E/G, SIGLEC 7/9), lymphodepletion regimens, and nanoparticle-based CAR gene delivery. UCAR-T, universal chimeric antigen receptor T cells; PBMC, peripheral blood mononuclear cells; CAR, chimeric antigen receptor; CRISPR-Cas, clustered regularly interspaced short palindromic repeats/CRISPR-associated protein 9; B2M, Beta-2 microglobulin; CIITA, class II major histocompatibility complex transactivator; RFX, regulatory factor X; shRNA, short hairpin RNA; NK, natural killer

The mechanism of UCAR-T cell therapy is fundamentally similar to that of conventional CAR-T cell therapy (Fig. 1), involving the genetic engineering of T cells to express a CAR that specifically recognizes TAAs [39–41]. However, the key distinction lies in the origin and availability of the T cells. In UCAR-T cell therapy, the T cells are derived from healthy donors and are genetically modified to prevent graft-vs.-host disease (GvHD) and host-vs.-graft (HvG) reactions, enabling their use in multiple patients without the need for individualized production. To generate UCAR-T cells, several gene-editing strategies, such as the disruption of the T-cell receptor (TCR) and human leukocyte antigen (HLA) class I molecules, are employed. This minimizes immune rejection and allows these allogeneic T cells to be administered as “off-the-shelf” products. One of the major advantages of UCAR-T therapy is the potential to streamline manufacturing, reduce treatment delays, and enhance accessibility, particularly for patients with rapidly progressing cancers or those lacking sufficient autologous T cells [42]. Additionally, it provides a scalable and economical approach to expanding global access to CAR-T cell therapy. In essence, UCAR-T therapy utilizes gene-edited allogeneic T cells engineered to express a single tumor-specific CAR, offering a universal and readily accessible immunotherapy alternative that circumvents the complexities associated with autologous cell preparation.

#### 2.2.2 Comparison of CAR-T and UCAR-T Cell Therapies

CAR-T cell therapy has demonstrated remarkable efficacy in treating hematologic malignancies such as B-ALL, diffuse large B-cell lymphoma (DLBCL), and multiple myeloma [10,43–45]. This approach involves collecting a patient’s own T cells, modifying them to express CARs that recognize specific tumor antigens, growing the cells *in vitro* and then administering them back to the patient. Despite its success, autologous CAR-T therapy has notable limitations, including high production costs, manufacturing delays, and reduced feasibility in heavily pretreated or immunocompromised patients. In contrast, UCAR-T cell therapy uses T cells from healthy donors that are genetically engineered and manufactured in advance (Table 1). These cells can be stored and administered to multiple patients as an off-the-shelf product, significantly reducing the time and resources required for individualized preparation. Moreover, gene-editing techniques help minimize immunologic complications by removing endogenous TCRs and HLA molecules. While current CAR-T therapies are mostly limited to hematologic cancers, UCAR-T therapies are being explored for both hematologic and solid tumors. However, further clinical validation is needed to address challenges such as persistence, efficacy in the tumor microenvironment, and long-term safety. In summary, although autologous CAR-T therapy has transformed the treatment landscape for certain blood cancers, UCAR-T therapy offers a promising universal alternative with improved scalability, faster availability, and the potential to expand access to CAR-T therapy for a wider patient population [46,47].

**Table 1:** Comparisons of CAR-T and UCAR-T therapy

	UCAR-T Therapy	CAR-T Therapy
**Manufacturing procedure**		
**Origin of T cells**	Healthy contributor	Patient (recipient)
Proliferation potential	High	Limited
Immediate availability	Standardized product	Patient-specific design, lacking broad off-the-shelf access
Capacity for phenotype-specific T-cell selection	Increased	Limited
Initial T-cell integrity	Increased	Low
Access timeframe	Easily accessible	Time-consuming, possible to manufacturing failure
Technical Complexity	High (gene editing is required to avoid rejection)	Medium (relying on patient cell collection)
**Intervention**		
Conditioning required	Augmented lymphodepletion	Standard lymphodepletion regimen
Treatment response rate	Medium	High
Range of indications	Wide	Restricted
*In vivo* persistence	Short	Long
**Side effects**		
GvHD	High risk	Low risk
Immunogenicity HvGR	Possible	Lower risk
CRS and ICANS	Yes	Yes
Cost	Lower	Expensive, upwards to US$350,000
**Advantages of clinical application**		
Applicable patients	Persons with low immune function or poor cell quality	Persons with normal immune function and healthy cells
Commercialization potential	High	Low

Note: CAR-T, chimeric antigen receptor-T; UCAR-T, universal chimeric antigen receptor-T; GvHD, graft-vs.-host disease; HvGR, host-vs.-graft rejection; CRS: Cytokine release syndrome; ICANS, immune effector cell-associated neurotoxicity syndrome.

## 3 Advances in UCAR-T Cell Therapy for Cancer Treatment

### 3.1 Engineering UCAR-T Cells

#### 3.1.1 Gene Editing Technologies

Gene editing technologies have revolutionized the field of CAR-T cell therapy, offering unprecedented opportunities to enhance the efficacy, safety, and accessibility of this innovative treatment. Among the various gene editing tools available, clustered regularly interspaced short palindromic repeat (CRISPR)/Cas9 has emerged as a game changer because of its high efficiency, specificity, and ease of use [48–50]. This technology enables precise modification of the T cell genome, allowing for the introduction of the desired genetic changes without the need for viral vectors, thereby reducing the risk of insertional mutagenesis and off-target effects [51]. CRISPR/Cas9 gene editing can be used to address several critical challenges in CAR-T cell therapy. For instance, it can be used to knock out genes encoding inhibitory receptors such as programmed death receptor 1 (PD-1) and cytotoxic T lymphocyte-associated antigen-4 (CTLA-4) [52,53], thereby enhancing the antitumor activity of CAR-T cells and mitigating the risk of immune exhaustion [54,55]. Using CRISPR/Cas9 to knockdown CD38, TCR, human leukocyte antigen (HLA)-I, and HLA-II genes, followed by lentiviral transduction, can enable lectin-like transcript 1 (LLT1) overexpression, enhance UCAR-T cell activity, and prevent allogeneic rejection, providing the necessary insights for the development of UCAR-T cell therapy [56]. In addition to CRISPR/Cas9, other gene editing tools such as transcription activator-like effector (TALE) nucleases (TALENs) and zinc-finger nucleases (ZFNs) have also been employed in CAR-T cell therapy [57,58]. Although less efficient and more complex than CRISPR/Cas9, these technologies remain valuable for enhancing the functionality of CAR-T cells. Gene editing as a whole has propelled significant progress in CAR-T cell therapy, offering innovative solutions to some of its most critical challenges. As these tools continue to evolve, further advances in the efficacy, safety, and accessibility of CAR-T treatments are anticipated, ultimately improving outcomes for cancer patients worldwide.

#### 3.1.2 Overcoming Alloreactivity

Alloreactivity, an immune response against foreign cells, is a significant hurdle in the development of UCAR-T cell therapy [59]. Unlike autologous CAR-T cells derived from a patient’s T cells, UCAR-T cells are designed as prebuilt products for use in multiple patients. This approach requires the use of allogeneic T cells, which can trigger an immune reaction against donor cells, leading to reduced therapeutic efficacy. Researchers have explored several strategies for addressing alloreactivity. One involves engineering UCAR-T cells to express costimulatory molecules, such as CD80 or CD40 ligands, enhancing the interactions between antigen-presenting cells (APCs) and T cells [60]. Additionally, targeting non-self-antigens with CARs minimizes alloreactivity risks because these antigens are less likely to be present in recipient cells [61]. Another approach modifies T cells to express inhibitory receptors such as PD-1 or CTLA-4, thereby dampening immune responses [62]. Preclinical research has demonstrated that engineered T cells display diminished alloreactivity alongside enhanced antitumor efficacy. Gene editing technologies, particularly CRISPR/Cas9, have been instrumental in achieving these modifications by precisely altering T cells to minimize immune rejection. For instance, CRISPR/Cas9 can knockout genes encoding alloreactive TCR or introduce genes encoding inhibitory receptors [63]. This innovation holds promise for the development of more robust UCAR-T cell therapies with fewer side effects. In summary, overcoming alloreactivity remains critical for advancing UCAR-T cell therapy. By leveraging strategies involving co-stimulatory molecules, inhibitory receptors, and gene editing technologies, researchers aim to enhance safety and efficacy in patients with cancer. These advancements underscore the transformative potential of combining CRISPR/Cas9 with CAR-T cell therapy in modern oncology [63].

### 3.2 Preclinical and Clinical Successes

#### 3.2.1 Preclinical Studies

Preclinical studies have played a pivotal role in the development of UCAR-T cell therapies. These studies provide valuable insights into the efficacy, safety, and feasibility of this innovative approach. One of the key preclinical studies involved the use of CRISPR/Cas9 technology to engineer UCAR-T cells. This technology has allowed researchers to precisely edit the genome of T cells by introducing desired genetic modifications without the need for viral vectors [64]. UCAR-T cell therapy faces the challenge of quickly clearing allogeneic cells from the host immune system. Another significant preclinical study focused on enhancing the antitumor activity of UCAR-T cells. For example, a study screened a safe and effective anti-CD70scFv for constructing anti-CD70CAR-T cells and then produced anti-CD70UCAR-T cells by knocking out the T cell receptor alpha constant (TRAC), beta 2-microglobulin (B2M) and human leukocyte antigen-DR alpha (HLA-DRA) [65]. To overcome the limitations of UCAR-T cell therapy, there is an “integrated” self-activation and protection module was developed for integration into CAR scaffolds. The SAP module comprises the CD47 extracellular domain, a mutated interleukin 7 receptor α (IL7Rα) transmembrane region, and its intracellular domain, designed to shield UCAR-T cells from host immune responses and improve their survival [65]. Furthermore, preclinical studies have investigated the application of UCAR-T cells in various cancer types. For instance, one study demonstrated the efficacy of UCAR-T cells in treating glioblastoma, a challenging solid tumor. By engineering CAR-T cells resistant to PD-1 inhibition, prolonged survival has been achieved in mice with intracranial tumors [66]. An experimental study developed a clinically feasible culture method to generate IL-15-expressing allogeneic CAR natural killer T (NKT) cells from human hematopoietic stem/progenitor cells (HSPCs), targeting seven types of cancer; a clinical trial was conducted to evaluate the efficacy of this method. These cells showed antitumor activity, expansion capacity, and persistence in multiple myeloma models [67]. These preclinical findings have laid the groundwork for the clinical translation of UCAR-T cell therapy, offering hope to patients with a wide range of malignancies. However, although preclinical studies have shown promising results, further research is needed to address the challenges associated with translating these findings into clinical practice.

#### 3.2.2 Clinical Trials of UCAR-T in Various Cancer Types

##### Hematological Malignancies

In recent years, advancements in gene editing technologies and fundamental cell biology have led to the exploration of various new approaches addressing the challenges mentioned above. Some of these novel discoveries have already progressed to the clinical stage, as outlined in the list of clinical trials of UCAR-T therapy for hematologic (Table 2) and solid (Table 3) tumors. Clinical trials are key contributors in advancing the development and validation of UCAR-T cell therapies. These trials were designed to assess the safety, efficacy, and optimal dose of the therapy in diverse patient populations. To date, several clinical trials have been conducted to evaluate the potential of UCAR-T cells for the treatment of various types of cancer. These trials have provided valuable insights into the safety, efficacy, and optimal dosing of the therapy, laying the groundwork for its further development (Table 2) and potential approval for clinical use. As research continues to advance, we expect to see more clinical trials exploring the potential of UCAR-T cell therapy for treating a wider range of cancer types.

**Table 2:** Clinical trials reporting results for UCAR-T therapy in hematologic malignancies

Drug name	Indication	Phase	Location	Strategies	Target	Safety assessment population	Efficacy assessment population	ORR N (%)	CR N (%)	References
ALLO-501	R/r B Lymphoma	I	USA	CD52 genes by TALENs and disruption of TRAC	CD19	46	32	24 (75)	16 (50)	[68]
ALLO-715	R/r multiple myeloma	I	USA	Lymphodeplet ion with an anti-CD52 antibody (ALLO-647)-containing regimen	BCMA	52	36	27 (75)	18 (50)	[69]
CYAD-211	R/r multiple myeloma	I	USA	Application of shRNA to knock down CD3ζ mRNA expression in TCR signaling	BCMA	9	9	2 (12.6)	_	[70]
CTX110	B-cell malignancies	I	USA	CRISPR/Cas9-mediated knockout of the TRAC gene	CD19	32	32	18 (56.3)	11 (34.4)	[71]
FT819	B-cell malignancies	I	USA	IPSc-derived T cells, disruption of the TRAC gene by CRISPR/Cas9	CD19	12	_	_	_	[72]
P-BCMA-ALL O1	Multiple myeloma	I	USA	Cas-CLOVER^TM^ gene editing used to disrupt TRBC and B2M genes.	BCMA	24	_	_	_	[73]
Nathali-01	R/r NHL	I	USA	TALEN-mediated inactivation of TRAC and CD52 genes.	CD20, CD22	3	3	3 (100)	2 (66.7)	[74]
CTX130	R/r T-cell lymphoma	I	USA, Australia, Canada	CRISPR/Cas9-mediated knockout of the TRAC gene	CD70	39	39	18 (46.2)	6 (19.4)	[75]
TruUCAR^TM^ GC502	R/r B-ALL	I	China	CD7 loci and TRAC were disrupted	CD19/CD7	4	4	4 (100)	3 (75)	[76]
UCART22	B-ALL	I process 2	USA	TALENs employed to disrupt the TRAC and CD52 genes.	CD22	3	3	2 (66.7)	1 (33.3)	[77]
ThisCART 19A	R/r B-ALL, relapsed NHL	I	China	Intracellular retention of TCR/HLA-I	CD19	8	7	_	7 (100)	[78]
UCART19	R/r B-ALL	I	USA	TALENs employed to disrupt the TRAC and CD52 genes.	CD19	21	21	_	14 (67)	[79]
PBCAR0191	B-ALL	I/II	USA	TRAC gene disrupted using the versatile ARCUS genome-editing platform	CD19	15	15	_	9 (60)	[80]
WU-CART-007	R/r TALL, r/r LBL	I	USA	CRISPR/Cas9-mediated disruption of CD7 and TRAC genes.	CD7	18	12	_	7 (58.3)	[81]
TT52CAR19	B-ALL	I	UK	CRISPR/Cas9-mediated disruption of CD52 and TRAC genes.	CD19	6	6	_	_	[82]
CTA101	R/r B-ALL	I	China	CD52 and TRAC knockout by Cas9	CD19/CD22	6	6	6 (100)	2 (33.3)	[83]
RD13-01	R/r TALL, r/r LBL	I	China	TCR/CD3 and CD7 knockout by CRISPR/Cas9	CD7	12	11	9 (88.8)	7 (63.6)	[84]
CARCIK-CD19	R/r B-ALL	I	Italy	Sleeping beauty transposon induction of CIK cells	CD19	13	13	_	8 (61.5)	[85]
SC291	NHL, CLL	I	US	Knockout of CD3 and HLA class I/II genes with CD47 overexpression.	CD19	1	_	_	_	[86]
AVC-101 (UniCAR02-T-CD123)	R/r AML	I	Germany	Adapter CAR-T consists of a UCAR-T cell and a CD123	CD123	19	15	8 (53)	_	[87]
CD33CART	R/r AML	I	USA	_	CD33	19	19	_	2 (11)	[88]
Base-Edited CAR7 T	Relapsed T-ALL	I	UK	CRISPR/nCas9 base editing	CD7	3	_	_	_	[89]
CLL-1 CAR-T	Relapsed AML	I	China	_	CD4/CD8	12	12	12 (100)	_	[90]
CAR EBV-VST	R/r B-cell malignancies	I	US	Modified with a CD19-specific CAR (19-28z)	CD19	16	16	_	_	[91]
GC007g	R/r B-ALL	I	China	_	CD19	12	9	9 (100)	7 (77.8)	[92]
ADI-001	B-cell lymphoma	I	USA	γδT	CD20	9	9	7 (78)	7 (78)	[93]
CAR EBV-CTL	R/r B-cell malignancies	I	USA	EBV virus-specific T cell	CD19	16	_	_	_	[94]
CD30 CAR EBVST cells	CD30-Positive Lymphoma	I/II	USA	EBV virus-specific T cell	CD30	14	14	9 (69.2)	5 (38.5)	[95]
CD19CAR/vi rus-specific T cells	CD19 + B cell malignancies	I	USA	Virus-specific T cells	CD19	6	6	2 (33.3)	2 (33.3)	[96]
RJMty19	R/r B-cell NHL	I	USA	DNT cells	CD19	12	5	3 (25)	1 (8.3)	[97]

Note: R/r: relapsed/refractory; B-ALL: B cell acute lymphoblastic leukemia; T-ALL: T cell acute lymphoblastic leukemia; LBL: lymphoblastic lymphoma; BCMA: B cell maturation antigen; NHL: non-Hodgkin’s lymphoma; CLL: chronic lymphocytic leukemia; CIK: cytokine-induced killer; AML: acute myeloid leukemia; EBV: Epstein-Barr virus; DNT: double-negative T cell; EBV-VSTs: Epstein-Barr virus-specific T cells; EBV-CTLs: EBV-specific cytotoxic T lymphocytes; TRAC: T cell receptor alpha constant; TRBC: T cell receptor constant β chain; ORR: overall response rate; CR: complete response.

**Table 3:** Clinical trial outcomes for UCAR therapy in solid tumors

Drug name	Indication	Phase	Location	Strategies	Target	Safety assessment population	Efficacy assessment population	ORR N (%)	CR N (%)	References
CYAD-101	mCRC	Phase 1	Belgium	Inhibit the TCR signaling pathway in donor T cells	NK	15	15	2 (13.33)	_	[98]
ALLO_GD2-CART01	R/r HR-NB	Phase 1	Italy	CD4 or CD8 central memory CAR-positive T cells dominate	GD2	5	5	3 (60)	2 (40)	[99]
CTX130	ccRCC	Phase 1	USA	Disruption of the TRAC gene by CRISPR/Cas9	CD70	16	16	_	1 (6.3)	[100]

Note: R/r, relapsed/refractory; mCRC, metastatic colorectal cancer; TCR, T cell receptor; HR-NB, high-risk neuroblastoma; ccRCC, clear cell renal cell carcinoma; UCAR, universal chimeric antigen receptor; TRAC, T cell receptor alpha constant; ORR, overall response rate; CR, complete response.

Hematological malignancies, including ALL, acute myeloid leukemia (AML), and lymphoma, have been the primary focus of CAR-T cell therapy because of their unmet medical needs and the significant advancements made in this field [64,101,102]. The advancement of UCAR-T cell therapy has further expanded the potential of this treatment modality for hematological malignancies. In these malignancies, the genetic and molecular heterogeneity of tumors poses a significant challenge for targeted therapies. CAR-T cell therapy, with its ability to target tumor-specific antigens, has shown promising results in the treatment of hematological malignancies [103,104].

Numerous therapies targeting CD19 or BCMA are being investigated for R/r B cell malignancies, including B cell lymphoma, B-ALL, and NHL. ALLO-501 [68], a CD19-targeted therapy utilizing TALEN-mediated disruption of TRAC and CD52 genes, demonstrated an ORR of 75% and a CR rate of 50% in 32 efficacy-assessed patients, with safety evaluated in 46 participants. Similarly, CTX110 [71], which employs CRISPR/Cas9 to disrupt the TRAC gene, reported a 56.3% ORR and 34.4% CR in 32 patients. UCART22 [77], targeting CD22 via TALEN-edited TRAC and CD52 inactivation, achieved a 66.7% ORR and 33.3% CR in a small cohort of three patients. Notably, TruUCAR^TM^ GC502 [76], developed in China to disrupt both TRAC and CD7 loci for dual targeting of CD19 and CD7, showed a 100% ORR and 75% CR in four patients. Additional China-developed therapies, such as CTA101 CRISPR/Cas9-mediated TRAC and CD52 knockout, and GC007g with CD19-targeted [81,83], achieved 100% ORR in six and nine patients, respectively, although CR rates varied from 33.3% to 77.8%. Other therapies, including FT819 and PBCAR0191, have shown preliminary safety but lack efficacy data [72,80]. Nathali-01, targeting CD20 and CD22 via TRAC/CD52 inactivation, reported a 100% ORR and 66.7% CR in three patients [74]. A phase I dose-escalation trial (NCT03774654) was conducted to evaluate the safety and preliminary efficacy of allogeneic CD19-specific CAR-NKT cells in patients with relapsed or refractory B-cell malignancies. This study used NKT cells from healthy donors, engineered to express CD19-specific CAR, IL-15, and downregulated β2-microglobulin and CD74 by shRNA to reduce the expression of HLA class I and II molecules. The effects of CD19-specific CAR, IL-15, and CD74 on the expression of HLA class I and II molecules, thereby reducing immunogenicity, were further investigated. Although no formal clinical studies on allogeneic CAR-NKT cells have been published, the ongoing studies described above demonstrate the potential of this therapy for the treatment of B-cell malignancies. More data on its safety and efficacy are expected in the future.

BCMA remains the central focus of r/r multiple myeloma therapy. ALLO-715 [69], combining lymphodepletion with the anti-CD52 antibody ALLO-647 achieved a 75% ORR and 50% CR in 36 efficacy-evaluated patients, with safety assessed in 52 participants. In contrast, CYAD-211 [70], which uses shRNA to silence CD3ζ mRNA in TCRs, reported a limited efficacy of 12.6% ORR and no CR in nine patients. P-BCMA-ALLO1 [73], leveraging the Cas-CLOVER™ system to disrupt T-cell receptor constant β chain (TRBC) and B2M genes, demonstrated safety in 24 patients but lacked efficacy outcomes. ADI-001 [93], a γδT cell therapy targeting CD20 in B cell lymphoma, achieved a 78% ORR and 78% CR in nine patients, suggesting potential cross-indication utility. Additionally, CAR Epstein-Barr virus (EBV)-specific T cells (EBV-VSTs) and CAR EBV-specific cytotoxic T lymphocytes (EBV-CTLs) [91,94], which utilize Epstein-Barr virus-specific T cells modified with CD19-targeted CARs, were safe in 16 patients but showed pending efficacy results. These findings underscore the need to optimize gene editing platforms, such as TALENs, CRISPR, and combination strategies, to enhance BCMA-directed responses.

For T cell malignancies, CTX130 [75], a CD70-targeted therapy using CRISPR/Cas9 to disrupt TRAC, reported a 46.2% ORR and 19.4% CR in 39 patients across the US, Australia, and Canada. WU-CART-007 [81], targeting CD7 and TRAC via CRISPR achieved a 58.3% CR in 12 patients with r/r T-ALL or lymphoblastic lymphoma. Base-edited CAR7 T cells [89], developed in the UK, inactivate CD52, CD7, and TCR genes via CRISPR/nCas9 base editing, although efficacy data remain unreported. For AML, CD33CART [88], a CD33-targeted therapy, demonstrated an 11% complete response rate in 19 patients.

CAR-T, targeting CD4 and CD8, achieved a 100% objective response rate in 12 patients, but lacked complete response data. AVC-101 [87], an adapter CAR-T system combining UCAR-T cells with a CD123-targeting module, exhibited a 53% objective response rate in 15 patients. Emerging strategies, such as CD30 CAR EBV-specific T cells achieving a 69.2% objective response rate and 38.5% CR rate in CD30-positive lymphoma, and CD19CAR/virus-specific T cells, demonstrating a 33.3% objective and CR rate in CD19-positive malignancies, highlight innovative approaches to enhance precision and safety [95]. Additionally, RD13-01 [84], a CRISPR-edited therapy disrupting CD7 and TCR/CD3 in T cell ALL or lymphoblastic lymphoma, reported an 88.8% objective response rate and 63.6% complete response rate in 11 patients, further underscoring the transformative potential of gene editing technologies in addressing T cell malignancies.

##### Solid Tumors

Solid tumors present a significant challenge for CAR T-cell therapy because of their complex and heterogeneous nature, including dense stroma, hypoxia, and multiple immune checkpoints. However, because of the intricate location of solid tumors, CAR-T cell therapy for solid tumors encounters numerous challenges such as a hostile tumor microenvironment, supra/exotumor toxicity, and unwanted antigen specificity [105]. Many strategies and methods have been attempted to overcome these obstacles, including knocking out PD-1 expression or secreting cytokines/chemokines to arm CAR-T cells, and using CAR-T cells in combination with other therapies [106–108]. Despite these efforts, CAR-T cells have not been approved for the treatment of solid tumors. Encouraging and optimistic, more than 40 clinical trials on CAR-T cell treatment of solid tumors have been registered in China alone [109]. To combat T cell dysfunction in the tumor microenvironment, CAR-T therapy has made progress in solid tumor treatment, in which CAR-T cells are metabolized by modifying them to secrete interleukin 10 (IL-10) to eradicate solid tumors and maintain immune protection. IL-10 secretion promotes the proliferation and effector function of CAR-T cells, resulting in the complete regression of solid tumors and metastatic cancers in various cancer types in homogeneous and xenograft mouse models (including colon, breast, melanoma, and pancreatic cancer). IL-10CAR-T cells also induce stem cell-like memory responses in lymphoid organs, providing lasting protection against tumor reattack [110].

Three UCAR-based investigational therapies have demonstrated different results in clinical trials targeting solid tumors. CYAD-101 [98], which targets NK cells by inhibiting the T-cell receptor signaling pathway in donor T cells, was evaluated in a Phase 1 trial involving 15 patients with unresectable metastatic colorectal cancer. The therapy demonstrated a 13.33% objective response rate, although no complete responses were reported. ALLO_GD2-CART01 [99] designed to target GD2 using CD4 or CD8 central memory CAR-positive T cells, achieved a 60% objective response rate and 40% complete response rate in a small cohort of five patients with relapsed/refractory high-risk neuroblastoma, highlighting its potential in pediatric solid tumors. CTX130 [100], which employs CRISPR/Cas9-mediated disruption of the TRAC gene to target CD70 in clear cell renal cell carcinoma, reported a 6.3% complete response rate among 16 patients in a Phase 1 trial, although objective response data were not disclosed. These results underscore the challenges and early promise of UCAR therapies for solid tumors, particularly for optimizing targeting strategies, enhancing response durability, and addressing the heterogeneity of solid tumor microenvironments. Further refinement of gene editing technologies and combination regimens may improve clinical outcomes in difficult-to-treat malignancies.

## 4 Primary Barriers in UCAR-T Cell Therapy

### 4.1 Challenge of Immune Rejection in Transplantation

#### 4.1.1 Graft-Versus-Host Disease (GvHD)

UCAR-T, as a “ready-to-use” cell therapy approach, is designed to address the prolonged manufacturing timelines and high costs associated with autologous CAR-T therapy. However, its allogeneic origin introduces GvHD as a significant safety concern. GvHD occurs when donor T cells mount an immune response against recipient tissues—a phenomenon commonly observed following allogeneic stem cell transplantation. In the context of UCAR-T therapy, donor T cells may recognize the recipient’s major histocompatibility complex (MHC) molecules through their TCRs, thereby initiating the GvHD response. Alloreactivity of αβ-TCRS is the main cause of GvHD development, and therefore, knockout of TCR genes in donor T cells is considered an effective strategy to reduce GvHD risk [111]. A retrospective study analyzed 25 patients undergoing hematopoietic stem cell allograft with CD19-directed CAR-T cell therapy, 11 of whom developed symptoms of suspected GvHD, and three patients (12%) were diagnosed with GvHD induced by CAR-T cell therapy [112]. This study underscores the risk of GvHD following CAR-T cell therapy, especially concerning donor compatibility and the design of the CAR construct. In a mouse model, allogeneic CD19 CAR-T cells initiate fatal GvHD in the presence of CD19-positive leukemia, whereas GvHD was not observed in the absence of leukemia [113]. This indicates that the presence of tumor antigens enhances CAR-T cell activity, thereby increasing the likelihood of GvHD.

#### 4.1.2 Host-Versus-Graft (HvG)

HvG is a key barrier to the efficacy and persistence of UCAR-T cell therapy. This mechanism involves the recognition and clearance of foreign CAR-T cells by the host immune system, leading to transient survival and reduced efficacy *in vivo* [114]. Allogeneic CAR-T cells expressing donor MHC molecules, particularly HLA-I and HLA-II, may be recognized as “non-self” by the host’s CD8^+^ and CD4^+^ T cells, triggering an immune response that results in the clearance of CAR-T cells. Even in partial HLA matching, minor antigenic differences can trigger immune rejection [114]. To reduce T cell-mediated rejection, researchers tried to reduce CAR-T cell surface HLA expression by knocking out genes such as β2-microglobulin. However, deletion of HLA-I may activate the host’s NK cells, which recognize the “missing self” signal and attack CAR-T cells [23]. The host may produce antibodies against donor HLA molecules, forming anti-donor-specific antibodies (DSA), which may lead to the clearance of CAR-T cells and limit subsequent cell therapy [115].

### 4.2 Safety Concerns

#### 4.2.1 Cytokine Release Syndrome

Cytokine release syndrome (CRS) remains a major safety challenge in UCAR-T therapy, particularly in hematological malignancies [116,117]. CRS occurs when CAR-T cells recognize and attack healthy cells, leading to the release of large amounts of cytokines, such as interleukin-2 (IL-2) and interferon-gamma (IFN-γ), into the bloodstream. CRS is the predominant adverse event associated with CAR T-cell immunotherapy and exhibits variable incidence rates across clinical studies. As evidenced by comprehensive trial data, this systemic inflammatory condition manifests in 42%–100% of treated patients, with severe presentations (grade ≥3) occurring in 0%–46% of cases. A meta-analysis of 2592 patients from 84 clinical investigations revealed an aggregate CRS-associated mortality rate of 1% [118] underscoring the generally manageable nature of this complication when employing appropriate interventions. Molecular pathogenesis involves a three-phase cascade: initial CAR T-cell activation induces bystander immune cell stimulation, particularly in the monocyte/macrophage lineage, followed by cytokine-driven endothelial activation [119]. Endothelial dysregulation ultimately mediates the characteristic hemodynamic instability and capillary leak syndrome observed in patients with severe CRS. Due to their allogeneic origin, UCAR-T cells may elicit a stronger immune response in the host, thereby increasing the risk of CRS. In addition, to reduce the risk of GvHD, UCAR-T cells often knock out TCR and HLA molecules through gene editing, which may affect their interaction with the host immune system and reduce the risk of GvHD, indirectly affecting the occurrence of CRS [120].

#### 4.2.2 Off-Target Effects

Off-target effects represent a significant challenge in UCAR-T cell therapy, particularly when considering the potential of these therapies for a broader range of cancer types [121]. The potential for off-target effects is heightened by the fact that many cancer antigens are also expressed on normal cells such as hematopoietic stem cells, endothelial cells, and epithelial cells. For example, CD19, a common target in B cell malignancies, is also expressed in some normal B cells, which can lead to the destruction of these cells, resulting in severe autoimmune reactions. Similarly, the CD133 antigen, which is often targeted in solid tumors, is also expressed on normal stem cells, raising concerns about the potential for off-target effects. Various strategies have been employed to mitigate the risk of off-target effects. CAR-T cells targeting CLL-1 or CD123 escape the off-target tumor effect and immunosuppressive tumor microenvironment and have good efficacy in AML [122]. Despite these efforts, off-target effects remain a significant concern in CAR-T cell therapy. Clinical trials have reported severe adverse events, such as encephalitis and myocarditis, which are believed to be related to off-target effects [123]. To address this challenge, ongoing research is focused on developing more sophisticated CAR constructs and manufacturing processes that minimize the risk of off-target effects while maintaining the efficacy of CAR-T cell therapy.

### 4.3 Regulatory and Ethical Issues

#### 4.3.1 Regulatory Hurdles

The path to developing and approving UCAR-T cell therapy has significant regulatory challenges that have hindered its widespread adoption. One of the primary obstacles is the lengthy and intricate approval process, which requires extensive preclinical and clinical data to establish safety and efficacy. This process becomes even more complex because of the autologous nature of UCAR-T cells, which are derived from the patient’s cells, and introduce additional variables and uncertainties into clinical trials. Regulatory bodies, such as the U.S. FDA and the European Medicines Agency (EMA), set rigorous criteria for the approval of cellular therapies, such as CAR-T cells [124,125]. These requirements include demonstrating the consistency and reproducibility of the manufacturing process, ensuring the quality and purity of the final product, and providing evidence of its long-term safety and efficacy. The unique characteristics of CAR-T cell therapy, particularly the use of gene editing technologies such as CRISPR/Cas9, further complicate the regulatory landscape. The potential for off-target effects and unintended genetic modifications necessitates a rigorous evaluation of the safety profile of the therapy, considering not only the risks associated with the gene editing process itself but also the possibility of modified cells causing harm to the patient. Collaboration between academic institutions, biotechnology companies, and collaboration with regulatory agencies is crucial for the effective development and approval of UCAR-T cell therapy. However, such collaboration introduces additional complexities and challenges. Coordinated approaches to data sharing, intellectual property rights, and clinical trial design are crucial but can slow down the approval process and increase costs. Addressing these challenges will be pivotal for the successful development and broad adoption of UCAR-T cell therapy as a cancer treatment. Despite these hurdles, ongoing advancements in gene editing technologies and innovative strategies to enhance the safety and efficacy of CAR-T cells offer hope for overcoming these barriers. By refining the manufacturing processes, improving target specificity, and reducing adverse effects, researchers aim to make UCAR-T cell therapy a more accessible and effective option for patients with cancer.

#### 4.3.2 Ethical Considerations

The ethical dimensions of UCAR-T cell therapy are complex and multifaceted. One primary concern is the potential for unintended consequences and off-target effects, particularly given the intricate nature of the human immune system and the implications of genetic modifications. There is a risk that these therapies could inadvertently trigger immune responses against healthy cells or tissues, leading to unforeseen complications. Ensuring the safety and efficacy of UCAR-T cells requires rigorous preclinical and clinical testing to address these risks. Another critical consideration pertains to equity and accessibility. The high cost of CAR-T cell therapy raises concerns that UCAR-T cells might become a luxury treatment accessible only to those with financial resources, potentially exacerbating existing health disparities. Efforts must be made to ensure affordability and accessibility for all patients in need, in line with the broader ethical principles of fairness and justice. Additionally, the use of gene editing technologies in developing UCAR-T cells introduces ethical questions regarding unintended genetic modifications and their long-term implications. The potential for heritable changes in the genome and unintended genetic drift necessitate careful oversight to ensure the responsible and ethical application of these technologies. This underscores the importance of robust regulatory frameworks and the ongoing evaluation of the safety and efficacy of gene editing tools. Finally, patient autonomy and informed consent are fundamental ethical considerations. Patients must be fully informed of the potential risks and benefits of UCAR-T cell therapy to make autonomous decisions regarding their treatment. Upholding these principles ensures that the development and implementation of UCAR-T cell therapy align with the highest ethical standards [126].

## 5 Future Directions and Perspectives

Improving the therapeutic effectiveness and safety profile of UCAR-T cell therapy remains a critical focus for advancing this innovative cancer treatment. Researchers are exploring multiple strategies to achieve these goals, starting with the optimization of the CAR structure. Fine-tuning of the antigen recognition domain can enhance specificity while minimizing off-target effects, which is crucial for improving therapeutic outcomes. Additionally, incorporating costimulatory domains boosts T cell activation and proliferation, leading to a more robust antitumor response. The development of bispecific CARs that simultaneously target multiple antigens represents another promising approach to increase the likelihood of successful tumor eradication. Addressing T cell exhaustion is vital for improving the persistence and overall effectiveness of CAR-T cells. This challenge can be addressed using various methods, such as engineering CAR-T cells to express inhibitory receptors capable of neutralizing signals from the immunosuppressive tumor microenvironment. Modulating the tumor microenvironment itself by reducing factors that suppress immunity further supports T cell activation. Improving the manufacturing process of UCAR-T cells is essential to reduce costs and ensure scalability. Innovations in production methods are also important. For example, constitutive expression of mutant B2M-HLA-E (MBE) and B2M-HLA-G (MBG) fusion proteins in anti-CD19 UCART (UCART-19) cells was carried out to prevent allogeneic NK cell-mediated lysis, and UCART-19 cells constitutively expressing MBE and MBG fusion proteins were created, showing effective and specific antitumor activity. Constitutive expression of MBE and MBG fusion proteins in UCART-19 cells prevents allogeneic NK cell-mediated lysis [127]. By focusing on these multifaceted strategies, researchers and clinicians aim to enhance both the efficacy and safety of UCAR-T cell therapy, making it a more accessible and effective treatment option for patients with cancer globally.

Expanding the application of UCAR-T cell therapy is essential for advancing cancer treatment. Although this therapy has primarily been applied to hematological malignancies such as ALL, NHL, and CLL, its potential extends far beyond these diseases [64,101,128]. Researchers are actively exploring the use of UCAR-T cells to treat solid tumors, which account for a significant proportion of cancer-related deaths. The challenges of applying CAR-T cells to solid tumors are complex. Solid tumors often exhibit heterogeneous microenvironments that hinder CAR T-cell infiltration and effectiveness. Moreover, immunosuppressive factors within the tumor microenvironment further compromise the efficacy of therapy. In recent years, UCAR-T therapy has made breakthroughs in the field of solid tumor treatment; however, it still faces multiple biological challenges. Unlike hematological tumors, factors such as the heterogeneity of solid tumors, immunosuppressive microenvironments (TMEs), and lack of ideal target antigens significantly limit the infiltration, activity, and durability of CAR-T cells. In response to these bottlenecks, researchers optimized UCAR-T design through multi-dimensional strategies: 1) target selection optimization: develop CAR structures for solid tumor-specific antigens or tumor microenvironment-related targets [99], and explore dual-target/logical gating CAR design to reduce off-target toxicity [129,130]; 2) microenvironment regulation: gene editing technology confers UCAR-T cells the ability to secrete immunomodulatory factors such as IL-12, PD-1 inhibitors [66], or knock out TGF-β receptors to resist TME inhibition; and 3) allogeneic rejection control: using gene editing to eliminate TCR and HLA molecule expression, and in combination with an immunosuppressive regimen to reduce the risk of GvHD [131]. In terms of clinical translation, early trials showed some responses. For example, UCAR-T cells targeting GD2 achieved objective remission in neuroblastoma (NCT04539366), whereas allogeneic CAR-T cells combined with an RNA vaccine against claudin-6 (CLDN6) showed initial antitumor activity in testicular cancer (NCT04503278). However, their efficacy is limited by insufficient CAR-T cell expansion and TME-mediated depletion. To improve its efficacy, researchers are exploring combined treatment strategies such as the use of checkpoint inhibitors [132], oncolytic viruses, or local radiotherapy to enhance tumor immunogenicity.

UCAR-T cell therapy was originally used to treat malignant tumors of the blood and has recently been used to treat autoimmune diseases. The mechanism mainly involves resetting the patient’s immune system by targeting and removing abnormally activated B cells. CRISPR-Cas9 gene editing technology was used to genetically engineer healthy donor-derived CD19-targeting CAR-T cells to address immune rejection, which is a promising strategy for the treatment of CD19-related diseases, leading to the development of a new generation of UCAR-T therapy and the successful treatment of one patient with refractory immune-mediated necrotizing myopathy and two patients with diffuse cutaneous systemic sclerosis [133]. During the 6-month follow-up after treatment, all three patients experienced deep remission of symptoms, significant improvement in the disease clinical response index score, and reversal of inflammation and organ fibrosis; there were no CRS or other serious adverse events.

As an important breakthrough in tumor immunotherapy, CAR-T cell therapy faces multidimensional equity challenges in its global promotion, forming a new pattern of health inequality. Current barriers to implementation are primarily economic, with personalized preparation processes resulting in single-treatment costs of up to US$400,000–500,000, resulting and less than 5% patient coverage in low- and middle-income countries (LMICs). Most (75%) of the world’s CAR-T production centers are in North and Western Europe, which highlights a serious imbalance in the regional distribution of resources. This dilemma is exacerbated by a lack of technological infrastructure, and developing countries generally lack standardized cold-chain logistics, quality control systems, and specialist medical teams; no indigenous CAR-T production facilities have been established in Africa. Fragmentation of the regulatory system is also a key constraint, with an average delay of 3–5 years for FDA-/EMA-approved products in LMICs. Approximately 80% of LMICs do not yet have a dedicated regulatory framework, leading to a lengthy and uncertain approval process. To overcome these bottlenecks, the international community is exploring solutions through technological innovation and collaborative models. In terms of technological cost reduction, UCAR-T eliminates the TCR/HLA-I gene through CRISPR editing technology, which reduces the production cost by 60%–70%. Automated closed manufacturing systems, such as CliniMACS Prodigy, reduce the lead time from 14 days to 7 days [134], significantly improving accessibility. Regional coordinated innovation is beginning to bear fruit, with companies such as India’s Bharat Biotech pushing for localized production through technology transfer through their Asian CAR-T hub programmer [135], The multi-country Procurement Consortium (MPPA) uses the model of the Global Alliance for Vaccine Immunization (Gavi) for central bargaining to reduce prices. At the policy level, the pay-for-effects model implemented by the UK NHS and the WHO-led stepped approval path provide operational access schemes for resource-limited areas.

The development of UCAR-T cell therapy represents a significant advancement in cancer immunotherapy. Although CAR-T cell therapy has revolutionized the treatment of hematological malignancies, its application to solid tumors remains challenging. The development of UCAR-T cells with the potential to target a wide range of cancer antigens offers a promising solution to this problem. Advancements in gene editing technologies such as CRISPR/Cas9 have facilitated the engineering of UCAR-T cells, overcoming the limitations of traditional CAR-T cell therapy. However, the development of UCAR-T cell therapies is challenging. Technical challenges such as manufacturing complexity and ensuring persistence and efficacy need to be addressed. Safety concerns, including CRS and off-target effects, should be carefully managed. Additionally, regulatory and ethical issues pose significant hurdles in the development and implementation of UCAR-T cell therapy. Despite these challenges, the potential of UCAR-T cell therapy for the treatment of various types of cancers cannot be overlooked. Future research should focus on enhancing the efficacy and safety of UCAR-T cells, expanding their application to a broader range of cancer types and ensuring equitable global access to this innovative therapy. By addressing these obstacles and progressing UCAR-T cell therapy, we can open the door to a new chapter in cancer treatment, bringing hope and enhancing patient outcomes globally.

In conclusion, while UCAR-T cell therapy offers a promising “off-the-shelf” alternative to autologous CAR-T cell approaches, its interactions within the complex immunological landscape of individual patients remain incompletely understood. Continued research is essential to elucidate the immunobiological dynamics between UCAR-T cells and the host immune system, and to refine gene-editing techniques and manufacturing protocols that ensure consistent safety and efficacy across diverse patient populations.

## Data Availability

Data sharing is not applicable to this article as no datasets were generated or analyzed during the current study.

## Glossary

CAR: Chimeric antigen receptor
UCAR: Universal CAR
CD52: Cluster of differentiation 52
Ig: Immunoglobulin
TCR: T cell receptors
HLA: Human leukocyte antigen
CRISPR/Cas: Clustered regularly interspaced short palindromic repeat/Associated system
MSLN: Mesothelin
MHC: Major histocompatibility complex
DNT: Double-negative T
NK: Natural killer
ALL: Acute lymphoblastic leukemia
NHL: Non-Hodgkin lymphoma
ORR: Overall response rate
CR: Complete response
TAA: Tumor-associated antigens
B-ALL: B-cell acute lymphoblastic leukemia
BCMA: B-cell maturation antigen
LBCL: Large B cell lymphoma
DLBCL: Diffuse large B-cell lymphoma
TALEN: Transcription activator-like effector nucleases
ZFNs: Zinc-finger nucleases
PD-1: Programmed death receptor 1
LLT1: Lectin-like transcript 1
CTLA-4: Cytotoxic T lymphocyte-associated antigen-4
TRAC: T cell receptor alpha constant
B2M: Beta 2-microglobulin
HLA-DRA: Human leukocyte antigen-DR alpha
IL7Rα: Interleukin 7 receptor α
R/r: Relapsed/refractory
mCRC: Metastatic colorectal cancer
HR-NB: High-risk neuroblastoma
T-ALL: T-cell acute lymphoblastic leukemia
LBL: Lymphoblastic lymphoma
CLL: Chronic lymphocytic leukemia
CIK: Cytokine-induced killer
AML: Acute myeloid leukemia
EBV: Epstein-Barr virus
EBV-VSTs: Epstein-Barr virus-specific T cells
EBV-CTLs: EBV-specific cytotoxic T lymphocytes
IL-10: Interleukin 10
IL-7: Interleukin 7
IL-15: Interleukin 15
NKT: Natural killer T
CRS: Cytokine release syndrome
MBE: B2M-HLA-E
MBG: B2M-HLA-G
CLDN6: Claudin 6
TME: Immunosuppressive microenvironment
B2M: Beta-2 microglobulin
HSPCs: Hematopoietic stem/progenitor cells
CIITA: Class II major histocompatibility complex transactivator;
RFX: Regulatory factor X
shRNA: Short hairpin RNA
NK: Natural killer
ccRCC: Clear cell renal cell carcinoma
r/r LBCL: Relapsed/refractory large B cell lymphoma
